# Electrochemical data of polypyridine complexes of Ru(II)

**DOI:** 10.1016/j.dib.2019.104759

**Published:** 2019-11-06

**Authors:** Deidré van der Westhuizen, Karel G. von Eschwege, Jeanet Conradie

**Affiliations:** Department of Chemistry, University of the Free State, PO Box 339, Bloemfontein, 9300, South Africa

**Keywords:** Ruthenium bipyridyl, Ruthenium phenanthroline, Photocatalyst, DSSC dye, Redox potential prediction

## Abstract

The data here-in presented is associated to the research article, Electrochemistry and spectroscopy of substituted [Ru(phen)_3_]^2+^ and [Ru(bpy)3]^2+^ complexes [1].

Redox data obtained from cyclic voltammetry experiments of the oxidation of Ru(II) to Ru(III) of thirteen Ru(II)-polypyridine complexes is presented in this data in brief article. Data is obtained from the cyclic voltammograms at scan rates of two orders of magnitude (0.05–5.00 Vs^−1^) under similar experimental conditions, namely in acetonitrile as solvent and tetrabutylammonium hexafluorophosphate as supporting electrolyte, and reported *versus* the redox couple of Fe(II) of ferrocene.

**Table:** Specifications Table

Subject area	*Chemistry*
More specific subject area	*Electrochemistry*
Type of data	*Table, text file, graph, figure*
How data was acquired	*BAS* 100B/W *electrochemical analyzer.*
Data format	*Raw and Analyzed.*
Experimental factors	*Synthesized samples were used. Degassed the solvent-electrolyte solution, in this case acetonitrile, in the electrochemical cell with Ar(g) for approximately* 10 min. *Sample addition to the acetonitrile-electrolyte solution and degassed for approximately 3 minutes.* *A blanket of Ar(g) was maintained in the cell for the duration of the electrochemical analysis.*
Experimental features	*Electrochemical analyses of all the samples were done in an electrochemical cell* (2mL)*, containing a glassy carbon working electrode, Pt reference electrode and a Pt auxiliary electrode.* *The electrochemical cell was connected to a BAS* 100 B/W *electrochemical analyzer and the obtained data was moved to Excel for data analysis and diagram preparation.*
Data source location	*Department of Chemistry, University of the Free State,* *Nelson Mandela street, Bloemfontein, South Africa.*
Data accessibility	*Data is with article.*
Related research article	*Deidré van der Westhuizen, Karel G. von Eschwege and Jeanet Conradie,* *Electrochemistry and spectroscopy of substituted [Ru(phen)*_*3*_*]*^*2+*^*and [Ru(bpy)*_*3*_*]*^*2+*^*complexes, Electrochimica Acta,*https://doi.org/10.1016/j.electacta.2019.07.051.

**Table:** 

**Value of the Data** • This data provide cyclic voltammograms of the Ru^II/III^ redox couple of thirteen polypyridine complexes of Ru(II). • This data provide electrochemical data of the Ru^II/III^ redox couple of thirteen polypyridine complexes of Ru(II) for scan rates 0.05–5.0 Vs^−1^. • This data illustrate the influence of differently functionalized polypyridine ligands on the ease of oxidation of Ru^II/III^ in thirteen Ru(II) polypyridine complexes. • Redox data of a complex is a key component in order to determine its suitability to be used as redox indicator, catalyst and photo-active mediator in dye-sensitized solar cells (DSSC) [2]

## 1 Data

**Fig. 1:**
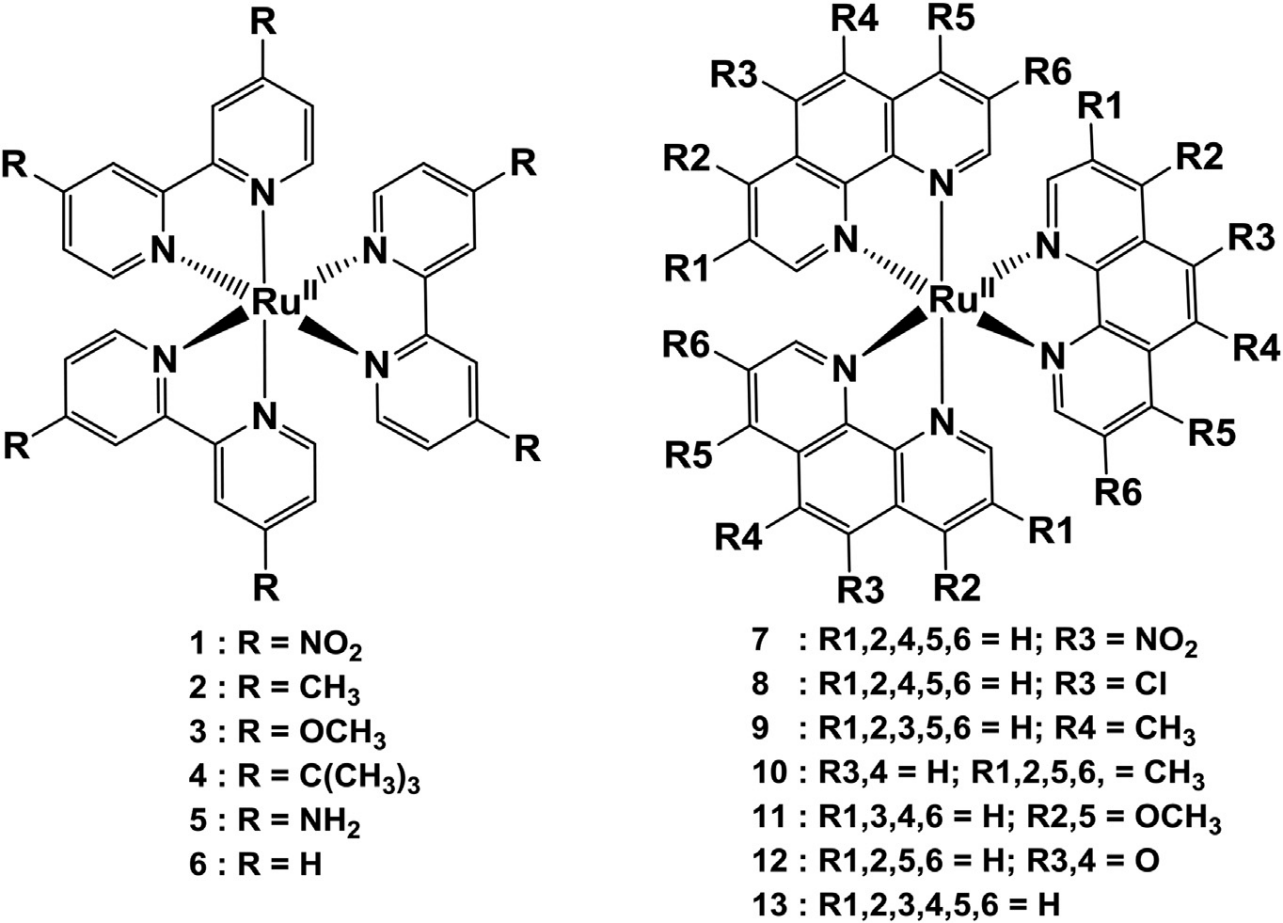
Structure and complex numbering of the polypyridine complexes of Ru(II).

**Fig. 2:**
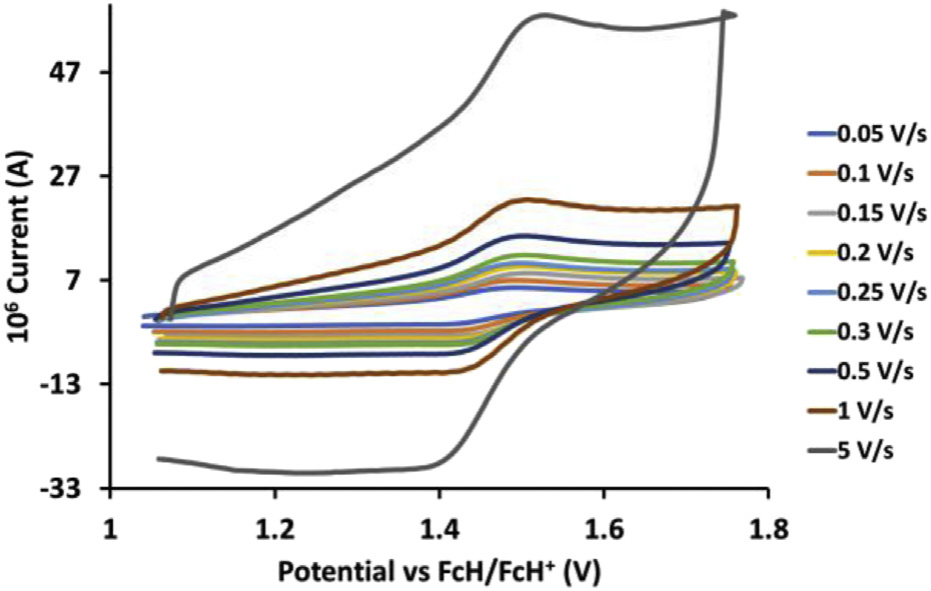
Cyclic voltammograms of *tris*(4,4′-dinitro-2,2′-bipyridine)ruthenium *bis*(tetrafluoroborate), 1, at scan rates 0.05 V/s to 5 V/s in the positive direction.

**Fig. 3:**
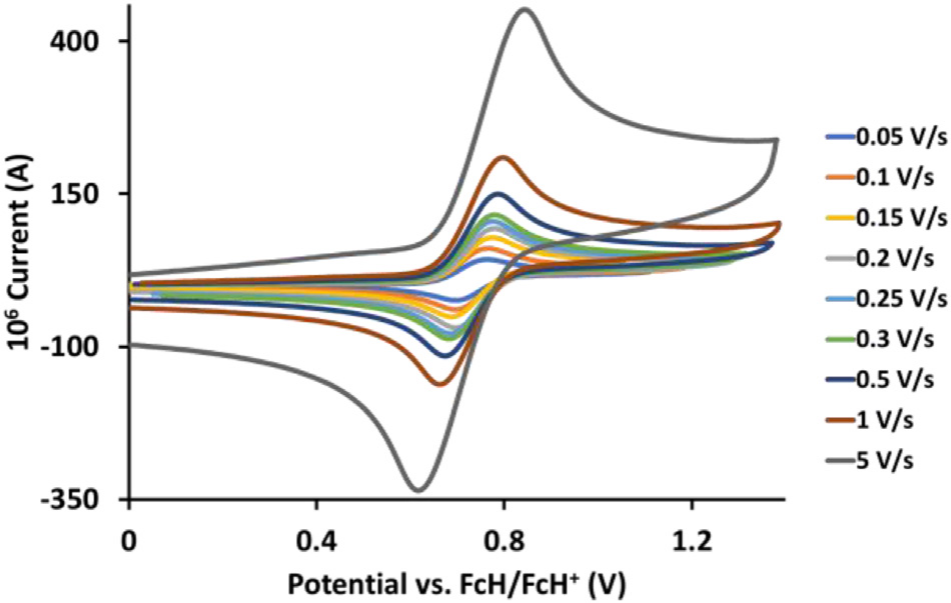
Cyclic voltammograms of *tris*(4,4′-dimethyl-2,2′-bipyridine)ruthenium *bis*(tetrafluoroborate), 2, at scan rates 0.05 V/s to 5 V/s in the positive direction.

**Fig. 4:**
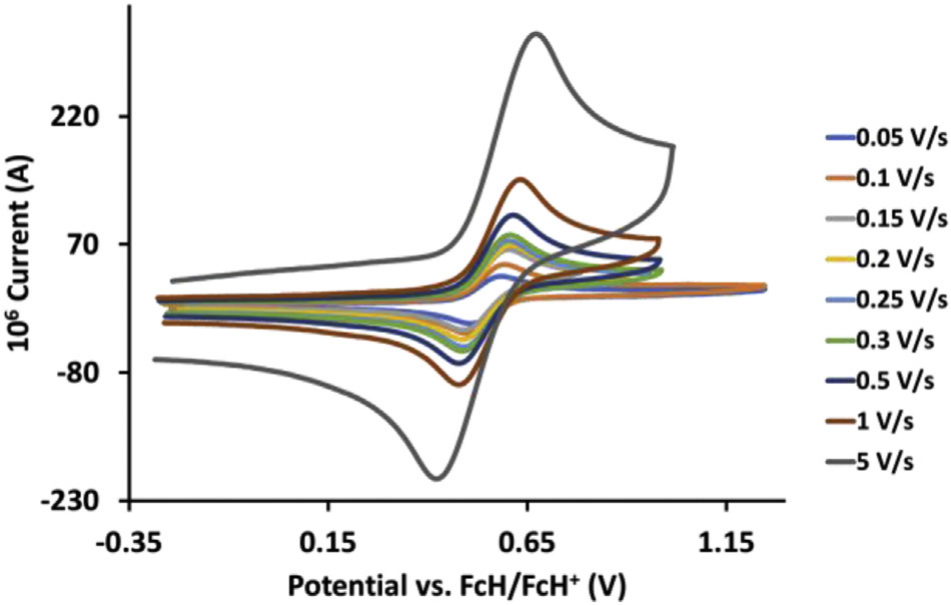
Cyclic voltammograms of *tris*(4,4′-dimethoxy-2,2′-bipyridine)ruthenium *bis*(tetrafluoroborate), 3, at scan rates 0.05 V/s to 5 V/s in the positive direction.

**Fig. 5:**
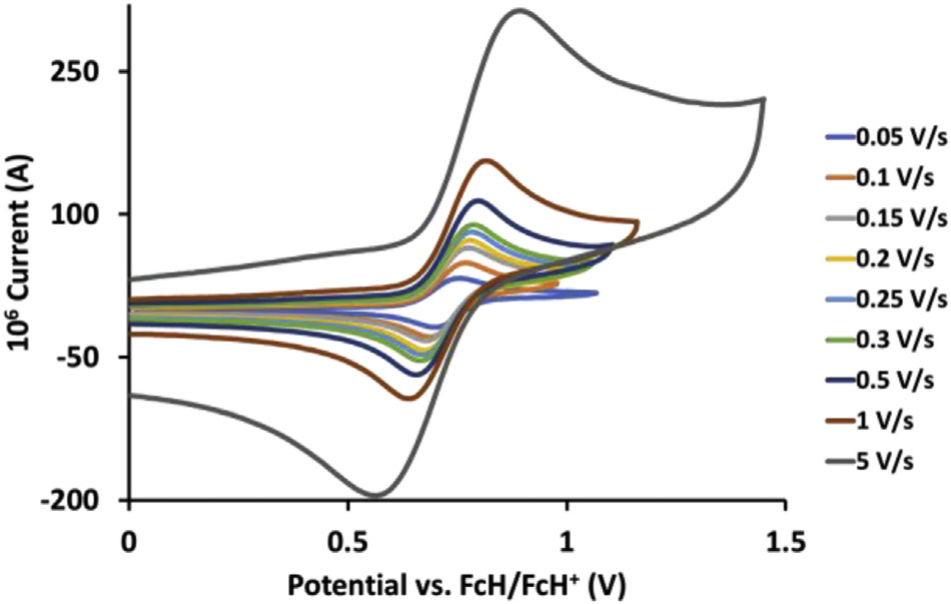
Cyclic voltammograms of *tris*(4,4′-di-*tert*-butyl-2,2′-bipyridine)ruthenium *bis*(tetrafluoroborate), 4, at scan rates 0.05 V/s to 5 V/s in the positive direction.

**Fig. 6:**
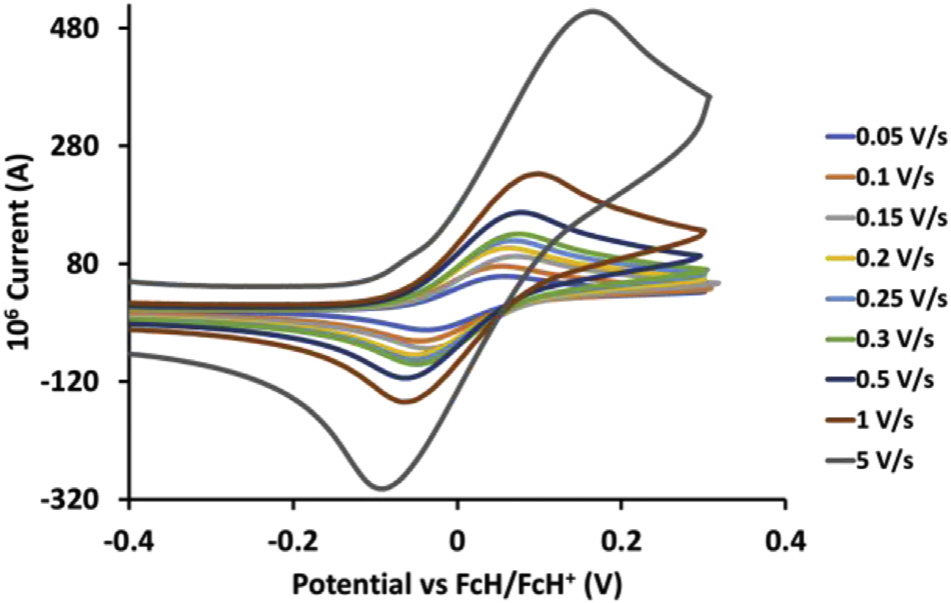
Cyclic voltammograms of *tris*(4,4′-diamino-2,2′-bipyridine)ruthenium *bis*(tetrafluoroborate), 5, at scan rates 0.05 V/s to 5 V/s in the positive direction.

**Fig. 7:**
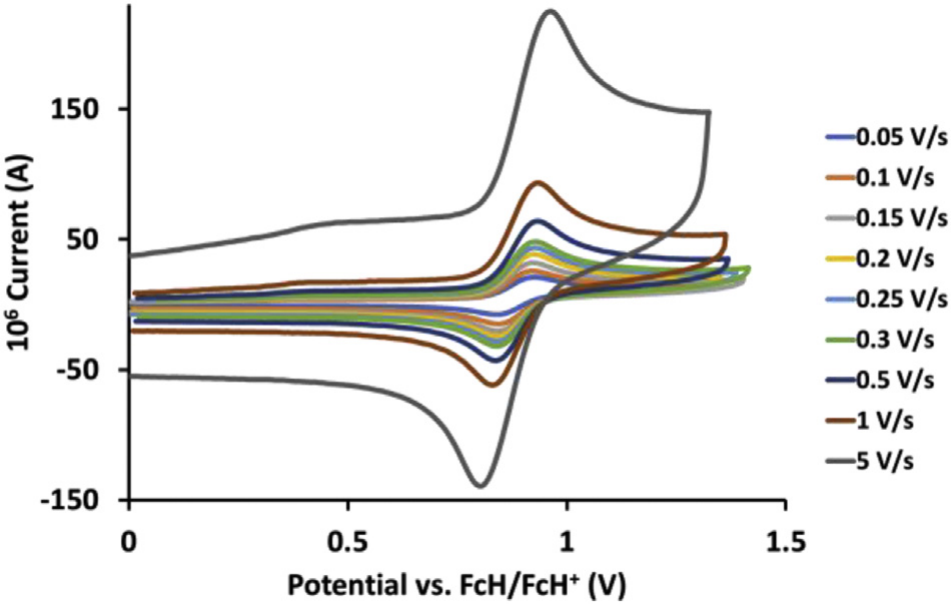
Cyclic voltammograms of *tris*(2,2′-bipyridine)ruthenium *bis*(tetrafluoroborate), 6, at scan rates 0.05 V/s to 5 V/s in the positive direction.

**Fig. 8:**
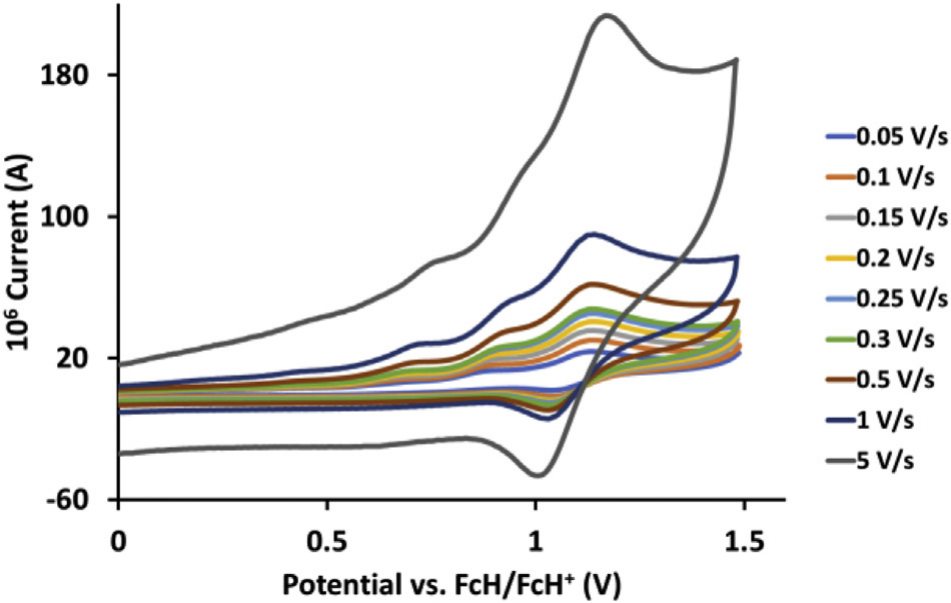
Cyclic voltammograms of *tris*(5-nitro-1,10-phenanthroline)ruthenium *bis*(tetrafluoroborate), 7, at scan rates 0.05 V/s to 5 V/s in the positive direction.

**Fig. 9:**
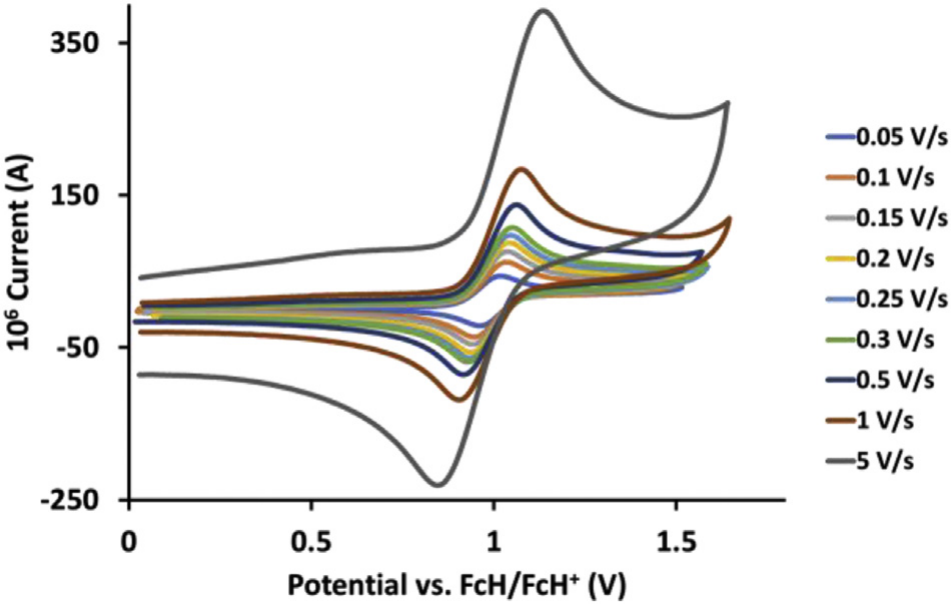
Cyclic voltammograms of *tris*(5-chloro-1,10-phenanthroline)ruthenium *bis*(tetrafluoroborate), 8, at scan rates 0.05 V/s to 5 V/s in the positive direction.

**Fig. 10:**
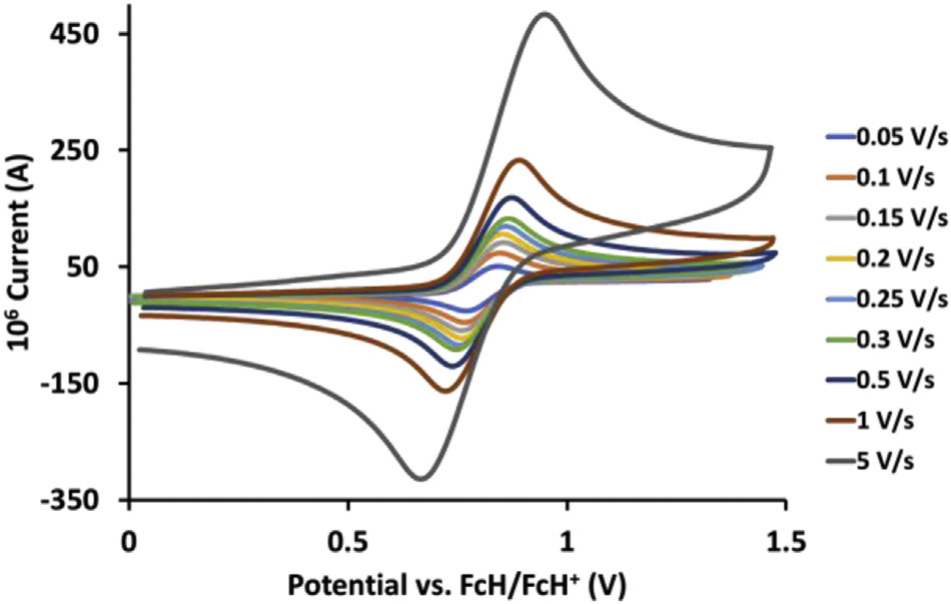
Cyclic voltammograms of *tris*(4-methyl-1,10-phenanthroline)ruthenium *bis*(tetrafluoroborate), 9, at scan rates 0.05 V/s to 5 V/s in the positive direction.

**Fig. 11:**
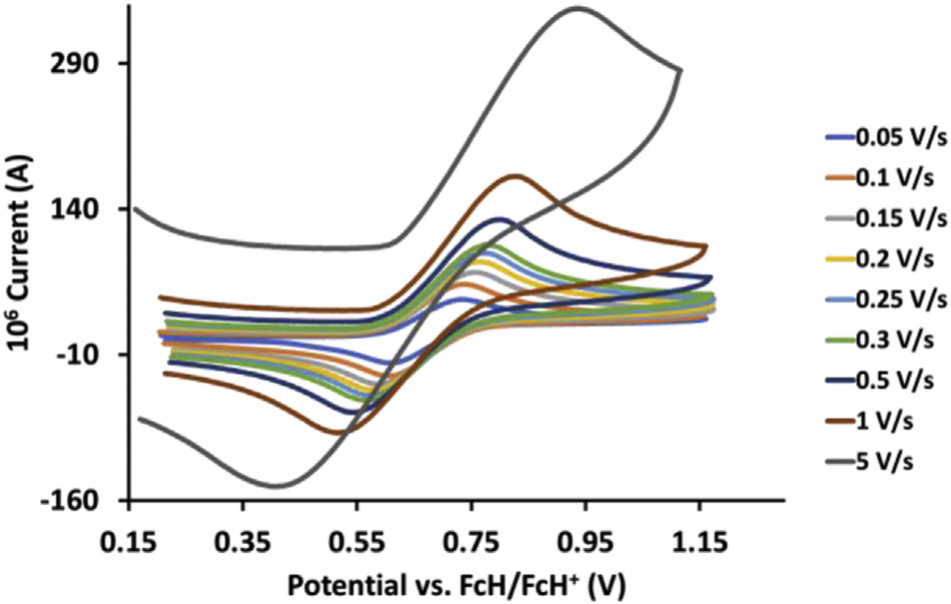
Cyclic voltammograms of *tris*(3,4,7,8-tetramethyl-1,10-phenanthroline)ruthenium *bis*(tetrafluoroborate), 10, at scan rates 0.05 V/s to 5 V/s in the positive direction.

**Fig. 12:**
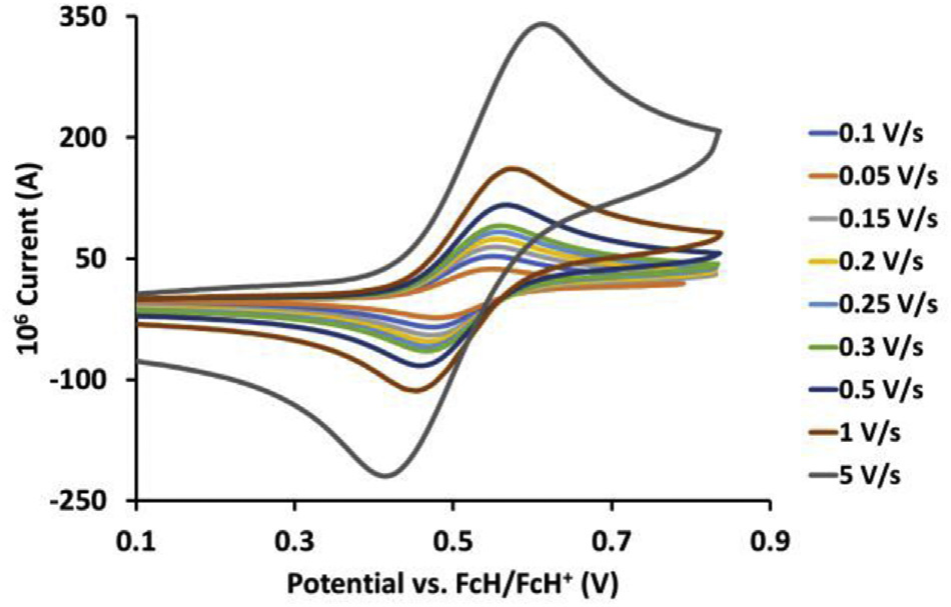
Cyclic voltammograms of *tris*(4,7-dimethoxy-1,10-phenanthroline)ruthenium *bis*(tetrafluoroborate), 11, at scan rates 0.05 V/s to 5 V/s in the positive direction.

**Fig. 13:**
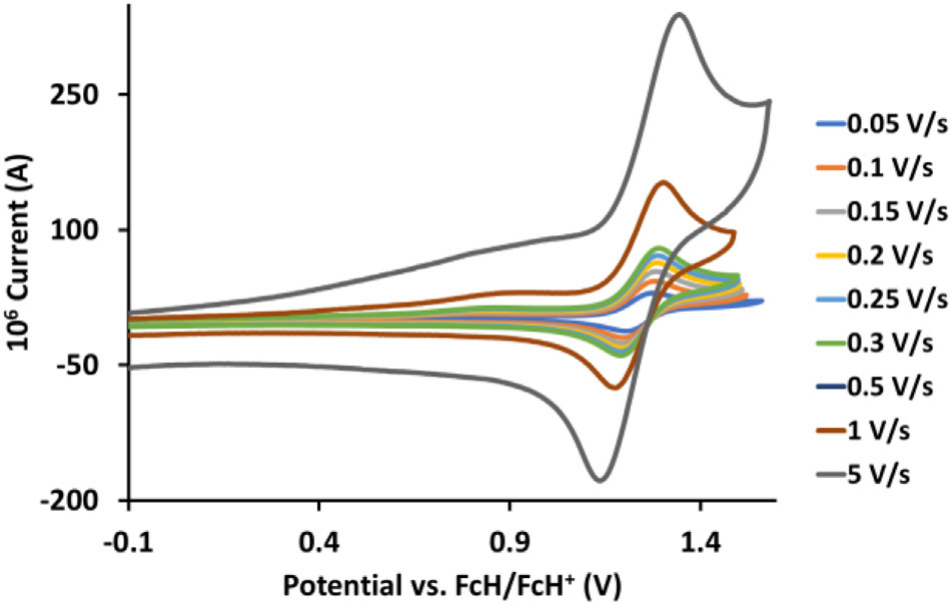
Cyclic voltammograms of *tris*(1,10-phenanthroline-5,6-dione)ruthenium(II) *bis*(hexafluorophosphate), 12, at scan rates 0.05 V/s to 5 V/s in the positive direction.

**Fig. 14:**
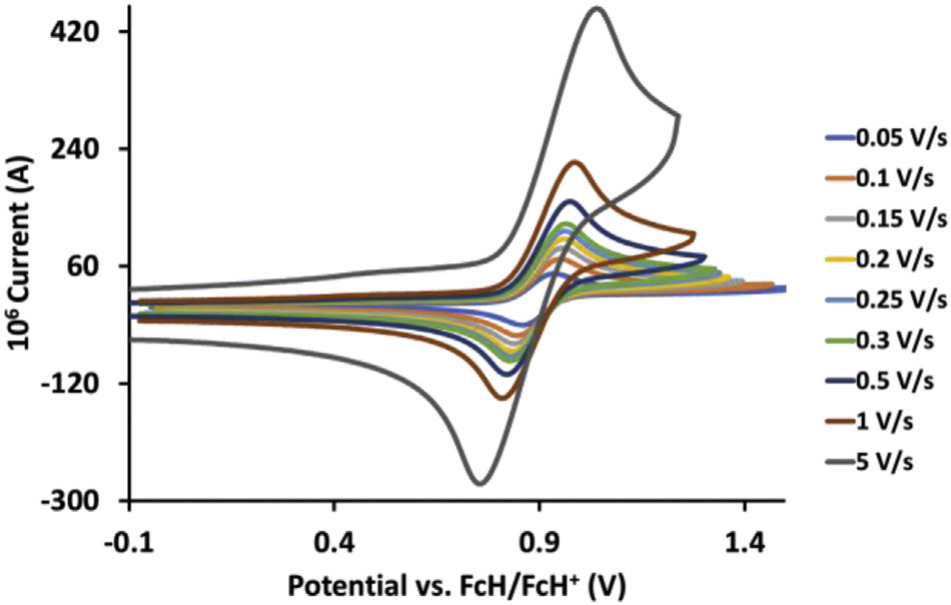
Cyclic voltammograms of *tris*(1,10-phenanthroline)ruthenium *bis*(tetrafluoroborate), 13, at scan rates 0.05 V/s to 5 V/s in the positive direction.

This article presents redox data of 13 octahedral Ru(II) complexes, 1–13, containing bipyridine-, substituted bipyridine-, phenanthroline- and substituted phenanthroline ligands, see Fig. 1 for the series of complexes of this data study. Presented data is related to the research article “Electrochemistry and spectroscopy of substituted [Ru(phen)_3_]^2+^ and [Ru(bpy)_3_]^2+^ complexes” [1]. Redox data of these Ru(II) complexes are important for application as redox indicators, catalysts and photo-active mediators in dye-sensitized solar cells (DSSC) [3,4]. Electrochemical data obtained from cyclic voltammograms at scan rates 0.05 Vs^−1^ – 5.00 Vs^−1^ (Fig. 2, Fig. 3, Fig. 4, Fig. 5, Fig. 6, Fig. 7, Fig. 8, Fig. 9, Fig. 10, Fig. 11, Fig. 12, Fig. 13, Fig. 14, 0.10 Vs^−1^ scans from Ref. [1]), are tabulated in Table 1, Table 2, Table 3, Table 4, Table 5, Table 6, Table 7, Table 8, Table 9, Table 10, Table 11, Table 12, Table 13.

**Table 1:** *Tris*(4,4′-dinitro-2,2′-bipyridine)ruthenium *bis*(tetrafluoroborate), 1, electrochemical data (potential in V vs. FcH) of the Ru(II/III) redox couple in acetonitrile (CH_3_CN) for *ca* 0.005 mol dm^−3^ complex solution at the indicated scan rates.

Scan Rate (V/s)	E_pa_(V)	10^6^ I_pa_ (A)	E_pc_ (V)	10^6^ I_pc_ (A)	E°’ (V)	ΔE(V)	I_pc_/I_pa_
0.05	1.493	1.430	1.417	1.403	1.455	0.076	0.98
0.10	1.495	2.506	1.414	2.453	1.455	0.081	0.98
0.15	1.507	3.015	1.403	2.949	1.455	0.104	0.98
0.20	1.503	3.301	1.407	3.275	1.455	0.096	0.99
0.25	1.501	3.436	1.408	3.361	1.455	0.093	0.98
0.30	1.505	4.128	1.404	4.032	1.455	0.101	0.98
0.50	1.506	5.405	1.404	5.277	1.455	0.102	0.98
1.00	1.507	8.137	1.403	7.934	1.455	0.104	0.98
5.00	1.515	17.39	1.394	16.99	1.455	0.121	0.98

**Table 2:** *Tris*(4,4′-dimethyl-2,2′-bipyridine)ruthenium *bis*(tetrafluoroborate), 2, electrochemical data (potential in V vs. FcH) of the Ru(II/III) redox couple in acetonitrile (CH_3_CN) for *ca* 0.005 mol dm^−3^ complex solution at the indicated scan rates.

Scan Rate (V/s)	E_pa_(V)	10^6^ I_pa_ (A)	E_pc_ (V)	10^6^ I_pc_ (A)	E^o^’ (V)	ΔE(V)	I_pc_/I_pa_
0.05	0.768	28.16	0.697	27.97	0.73	0.071	0.99
0.10	0.765	48.42	0.696	47.98	0.73	0.069	0.99
0.15	0.777	61.86	0.673	61.52	0.73	0.104	0.99
0.20	0.780	86.54	0.681	85.97	0.73	0.099	0.99
0.25	0.772	78.45	0.684	77.79	0.73	0.088	0.99
0.30	0.778	85.69	0.682	84.98	0.73	0.096	0.99
0.50	0.787	110.59	0.674	109.95	0.73	0.113	0.99
1.00	0.798	158.36	0.663	157.53	0.73	0.135	0.99
5.00	0.843	313.87	0.617	312.23	0.73	0.226	0.99

**Table 3:** *Tris*(4,4′-dimethoxy-2,2′-bipyridine)ruthenium *bis*(tetrafluoroborate), 3, electrochemical data (potential in V vs. FcH) of the Ru(II/III) redox couple in acetonitrile (CH_3_CN) for *ca* 0.005 mol dm^−3^ complex solution at the indicated scan rates.

Scan Rate (V/s)	E_pa_(V)	10^6^ I_pa_ (A)	E_pc_ (V)	10^6^ I_pc_ (A)	E^o^’ (V)	ΔE(V)	I_pc_/I_pa_
0.05	0.587	24.21	0.507	23.98	0.547	0.080	0.99
0.10	0.593	33.13	0.501	32.96	0.547	0.092	0.99
0.15	0.603	46.14	0.491	45.83	0.547	0.112	0.99
0.20	0.608	45.36	0.487	44.98	0.547	0.121	0.99
0.25	0.605	53.49	0.489	52.98	0.547	0.116	0.99
0.30	0.606	61.48	0.489	60.97	0.547	0.117	0.99
0.50	0.615	68.55	0.480	67.98	0.547	0.135	0.99
1.00	0.625	72.23	0.470	71.68	0.547	0.155	0.99
5.00	0.672	136.41	0.422	135.69	0.547	0.250	0.99

**Table 4:** *Tris*(4,4′-di-*tert*-butyl-2,2′-bipyridine)ruthenium *bis*(tetrafluoroborate), 4, electrochemical data (potential in V vs. FcH) of the Ru(II/III) redox couple in acetonitrile (CH_3_CN) for *ca* 0.005 mol dm^−3^ complex solution at the indicated scan rates.

Scan Rate (V/s)	E_pa_(V)	10^6^ I_pa_ (A)	E_pc_ (V)	10^6^ I_pc_ (A)	E^o^’ (V)	ΔE(V)	I_pc_/I_pa_
0.05	0.756	19.65	0.698	19.49	0.727	0.058	0.99
0.10	0.768	25.21	0.687	24.98	0.727	0.081	0.99
0.15	0.776	41.21	0.679	40.98	0.727	0.097	0.99
0.20	0.778	45.55	0.676	45.09	0.727	0.102	0.99
0.25	0.783	55.36	0.671	54.97	0.727	0.112	0.99
0.30	0.788	65.32	0.667	64.98	0.727	0.121	0.99
0.50	0.798	70.01	0.657	69.32	0.727	0.141	0.99
1.00	0.815	91.35	0.639	90.65	0.727	0.176	0.99
5.00	0.893	152.36	0.561	151.38	0.727	0.332	0.99

**Table 5:** *Tris*(4,4′-diamino-2,2′-bipyridine)ruthenium *bis*(tetrafluoroborate), 5, electrochemical data (potential in V vs. FcH) of the Ru(II/III) redox couple in acetonitrile (CH_3_CN) for *ca* 0.005 mol dm^−3^ complex solution at the indicated scan rates.

Scan Rate (V/s)	E_pa_(V)	10^6^ I_pa_ (A)	E_pc_ (V)	10^6^ I_pc_ (A)	E^o^’ (V)	ΔE(V)	I_pc_/I_pa_
0.05	0.054	35.31	−0.042	34.98	0.006	0.096	0.99
0.10	0.057	55.88	−0.045	55.34	0.006	0.102	0.99
0.15	0.059	74.15	−0.047	73.56	0.006	0.106	0.99
0.20	0.062	81.36	−0.051	80.89	0.006	0.113	0.99
0.25	0.066	95.32	−0.054	94.76	0.006	0.119	0.99
0.30	0.068	110.16	−0.056	109.58	0.006	0.124	0.99
0.50	0.076	117.35	−0.065	116.47	0.006	0.140	0.99
1.00	0.083	155.08	−0.071	154.12	0.006	0.154	0.99
5.00	0.135	301.75	−0.122	300.03	0.006	0.257	0.99

**Table 6:** *Tris*(2,2′-bipyridine)ruthenium *bis*(tetrafluoroborate), 6, electrochemical data (potential in V vs. FcH) of the Ru(II/III) redox couple in acetonitrile (CH_3_CN) for *ca* 0.005 mol dm^−3^ complex solution at the indicated scan rates.

Scan Rate (V/s)	E_pa_(V)	I_pa_ (μA)	E_pc_ (V)	I_pc_ (μA)	E^o^’ (V)	ΔE (V)	I_pc_/I_pa_
0.05	0.926	9.62	0.841	9.53	0.883	0.085	0.99
0.10	0.923	16.23	0.844	16.10	0.883	0.079	0.99
0.15	0.923	23.45	0.843	23.25	0.883	0.080	0.99
0.20	0.927	22.67	0.839	22.46	0.883	0.088	0.99
0.25	0.926	28.03	0.840	27.75	0.883	0.086	0.99
0.30	0.928	32.65	0.839	32.32	0.883	0.089	0.99
0.50	0.930	45.31	0.836	44.86	0.883	0.094	0.99
1.00	0.936	59.36	0.831	58.97	0.883	0.105	0.99
5.00	0.963	113.42	0.804	112.34	0.883	0.159	0.99

**Table 7:** *Tris*(5-nitro-1,10-phenanthroline)ruthenium, 7, *bis*(tetrafluoroborate) electrochemical data (potential in V vs. FcH) of the Ru(II/III) redox couple in acetonitrile (CH_3_CN) for *ca* 0.005 mol dm^−3^ complex solution at the indicated scan rates.

Scan Rate (V/s)	E_pa_(V)	I_pa_ (μA)	E_pc_ (V)	I_pc_ (μA)	E^o^’ (V)	ΔE (V)	I_pc_/I_pa_
0.05	1.132	5.9	1.04	4.1	1.085	0.093	0.70
0.10	1.130	10.2	1.04	9.4	1.085	0.090	0.92
0.15	1.133	14.9	1.04	12.7	1.085	0.096	0.85
0.20	1.134	13.1	1.04	12.0	1.085	0.097	0.92
0.25	1.137	22.2	1.03	18.8	1.085	0.104	0.85
0.30	1.136	29.0	1.03	24.0	1.085	0.103	0.83
0.50	1.140	35.2	1.03	29.1	1.085	0.110	0.83
1.00	1.140	43.9	1.03	36.4	1.085	0.111	0.83
5.00	1.169	112.9	1.00	98.7	1.085	0.168	0.87

**Table 8:** *Tris*(5-chloro-1,10-phenanthroline)ruthenium *bis*(tetrafluoroborate), 8, electrochemical data (potential in V vs. FcH) of the Ru(II/III) redox couple in acetonitrile (CH_3_CN) for *ca* 0.005 mol dm^−3^ complex solution at the indicated scan rates.

Scan Rate (V/s)	E_pa_(V)	I_pa_ (μA)	E_pc_ (V)	I_pc_ (μA)	E^o^’ (V)	ΔE (V)	I_pc_/I_pa_
0.05	1.02	28.66	0.961	28.47	0.99	0.058	0.99
0.10	1.04	44.50	0.945	44.23	0.99	0.90	0.99
0.15	1.04	53.93	0.942	53.87	0.99	0.096	1.00
0.20	1.04	57.96	0.937	57.54	0.99	0.106	0.99
0.25	1.05	70.25	0.934	69.65	0.99	0.113	0.99
0.30	1.05	66.21	0.929	65.75	0.99	0.122	0.99
0.50	1.06	85.23	0.918	84.65	0.99	0.145	0.99
1.00	1.07	134.21	0.906	133.65	0.99	0.169	1.00
5.00	1.13	279.12	0.846	276.34	0.99	0.289	0.99

**Table 9:** *Tris*(4-methyl-1,10-phenanthroline)ruthenium *bis*(tetrafluoroborate), 9, electrochemical data (potential in V vs. FcH) of the Ru(II/III) redox couple in acetonitrile (CH_3_CN) for *ca* 0.005 mol dm^−3^ complex solution at the indicated scan rates.

Scan Rate (V/s)	E_pa_(V)	10^6^ I_pa_ (A)	E_pc_ (V)	10^6^ I_pc_ (A)	E^o^’ (V)	ΔE (V)	Ipc/Ipa
0.05	0.844	42.15	0.771	41.74	0.807	0.073	0.99
0.10	0.847	55.32	0.768	54.95	0.807	0.079	0.99
0.15	0.854	74.41	0.760	73.85	0.807	0.094	0.99
0.20	0.854	82.05	0.761	81.76	0.807	0.093	1.00
0.25	0.861	91.39	0.754	90.77	0.807	0.107	0.99
0.30	0.866	92.62	0.748	91.98	0.807	0.118	0.99
0.50	0.875	128.43	0.740	127.23	0.807	0.135	0.99
1.00	0.891	161.65	0.723	160.23	0.807	0.168	0.99
5.00	0.949	316.17	0.665	314.77	0.807	0.284	1.00

**Table 10:** *Tris*(3,4,7,8-tetramethyl-1,10-phenanthroline)ruthenium *bis*(tetrafluoroborate), 10, electrochemical data (potential in V vs. FcH) of the Ru(II/III) redox couple in acetonitrile (CH_3_CN) for *ca* 0.005 mol dm^−3^ complex solution at the indicated scan rates.

Scan Rate (V/s)	E_pa_(V)	I_pa_ (μA)	E_pc_ (V)	I_pc_ (μA)	E^o^’ (V)	ΔE (V)	I_pc_/I_pa_
0.05	0.735	33.8	0.608	33.8	0.671	0.127	1.00
0.10	0.737	47.5	0.605	47.3	0.671	0.132	1.00
0.15	0.756	55.8	0.587	55.7	0.671	0.169	1.00
0.20	0.763	61.1	0.578	60.9	0.671	0.185	1.00
0.25	0.771	72.2	0.572	71.7	0.671	0.199	0.99
0.30	0.775	74.5	0.567	73.9	0.671	0.208	0.99
0.50	0.801	93.9	0.542	93.0	0.671	0.259	0.99
1.00	0.826	115.6	0.517	115.0	0.671	0.309	1.00
5.00	0.936	168.5	0.406	167.3	0.671	0.530	0.99

**Table 11:** *Tris*(4,7-dimethoxy-1,10-phenanthroline)ruthenium *bis*(tetrafluoroborate), 11, electrochemical data (potential in V vs. FcH) of the Ru(II/III) redox couple in acetonitrile (CH_3_CN) for *ca* 0.005 mol dm^−3^ complex solution at the indicated scan rates.

Scan Rate (V/s)	E_pa_(V)	10^6^ I_pa_ (A)	E_pc_ (V)	10^6^ I_pc_ (A)	E^o^’ (V)	ΔE (V)	Ipc/Ipa
0.05	0.548	29.5	0.48	29.2	0.513	0.069	0.99
0.10	0.551	41.4	0.48	40.9	0.513	0.076	0.99
0.15	0.554	49.4	0.47	48.9	0.513	0.081	0.99
0.20	0.555	59.2	0.47	58.5	0.513	0.084	0.99
0.25	0.556	67.9	0.47	66.9	0.513	0.087	0.99
0.30	0.560	75.8	0.47	74.9	0.513	0.094	0.99
0.50	0.568	105.0	0.46	103.9	0.513	0.110	0.99
1.00	0.573	140.7	0.45	138.9	0.513	0.120	0.99
5.00	0.613	253.6	0.41	250.0	0.513	0.199	0.99

**Table 12:** *Tris*(1,10-phenanthroline-5,6-dione)ruthenium(II) *bis*(hexafluorophosphate), 12, electrochemical data (potential in V vs. FcH) of the Ru(II/III) redox couple in acetonitrile (CH_3_CN) for *ca* 0.005 mol dm^−3^ complex solution at the indicated scan rates.

Scan Rate (V/s)	E_pa_(V)	10^6^ I_pa_ (A)	E_pc_ (V)	10^6^ I_pc_ (A)	E^o^’ (V)	ΔE(V)	Ipc/Ipa
0.05	1.276	22.1	1.20	21.9	1.240	0.071	0.99
0.10	1.280	31.2	1.20	30.8	1.240	0.080	0.99
0.15	1.283	42.2	1.20	41.5	1.240	0.087	0.99
0.20	1.286	47.4	1.19	46.8	1.240	0.092	0.99
0.25	1.287	52.7	1.19	51.9	1.240	0.094	0.99
0.30	1.291	57.6	1.19	56.9	1.240	0.101	0.99
0.50	1.296	79.1	1.18	78.0	1.240	0.112	0.99
1.00	1.302	113.2	1.18	112.0	1.240	0.125	0.99
5.00	1.344	221.6	1.14	218.3	1.240	0.208	0.99

**Table 13:** *Tris*(1,10-phenanthroline)ruthenium *bis*(tetrafluoroborate), 13, electrochemical data (potential in V vs. FcH) of the Ru(II/III) redox couple in acetonitrile (CH_3_CN) for *ca* 0.005 mol dm^−3^ complex solution at the indicated scan rates.

Scan Rate (V/s)	E_pa_(V)	10^6^ I_pa_ (A)	E_pc_ (V)	10^6^ I_pc_ (A)	E^o^’ (V)	ΔE (V)	Ipc/Ipa
0.05	0.936	42.03	0.858	41.65	0.897	0.078	0.99
0.10	0.950	64.12	0.844	64.03	0.897	0.106	1.00
0.15	0.957	83.14	0.838	82.56	0.897	0.119	0.99
0.20	0.960	94.65	0.835	93.98	0.897	0.125	0.99
0.25	0.962	102.36	0.833	101.95	0.897	0.129	1.00
0.30	0.966	103.94	0.829	102.98	0.897	0.137	0.99
0.50	0.974	126.48	0.821	125.65	0.897	0.153	0.99
1.00	0.985	178.65	0.809	176.98	0.897	0.176	0.99
5.00	1.040	312.32	0.755	309.97	0.897	0.285	0.99

## 2 Experimental design, materials, and methods

Electrochemical studies utilizing cyclic voltammetric measurements were done on a BAS100B Electrochemical Analyzer linked to a personal computer, utilizing BAS100W Version 2.3 software. Measurements were done at 293 K. Successive experiments under the same experimental conditions showed that all formal reduction and oxidation potentials were reproducible within 0.005 V. Cyclic voltammetric measurements were performed on 0.005 mol dm^−3^ solutions of the complex, dissolved in CH_3_CN, containing 0.200 mol dm^−3^ tetrabutylammonium hexafluorophosphate (TBAPF_6_, [NBu_4_][PF_6_]) as supporting electrolyte. Measurements were conducted under a blanket of purified Argon. A three-electrode cell consisting of a Pt auxiliary electrode, a glassy carbon (surface area 3.14 × 10^−6^ m^2^) working electrode and a Pt-wire pseudo reference electrode were used. The working electrode was polished on a Buhler polishing mat; first with 1 μm and lastly with ¼ micron diamond paste. Scan rates were between 0.05 and 5.00 V s^−1^. All experimental potentials were referenced against the redox couple of ferrocene FcH/FcH^+^ (IUPAC) [5].

## Conflict of Interest

The authors declare that they have no known competing financial interests or personal relationships that could have appeared to influence the work reported in this paper.
